# Correlation Between Mothers’ Depression and Developmental Delay in Infants Aged 6-18 Months

**DOI:** 10.5539/gjhs.v8n5p11

**Published:** 2015-08-20

**Authors:** Roshanak Vameghi, Sedigheh Amir Ali Akbari, Homeira Sajjadi, Firoozeh Sajedi, Hamid Alavimajd

**Affiliations:** 1 Pediatric Neurorehabilitation Research Center, University of Social Welfare and Rehabilitation Sciences, Tehran, Iran; 2 Social Determinants of Health Research Center, University of Social Welfare and Rehabilitation Sciences, Tehran, Iran; 3 Departments of Biostatistics, School of Paramedical Sciences, Shahid Beheshti University of Medical Sciences, Tehran, Iran

**Keywords:** Ages and Stages Questionnaire, developmental delay, depression

## Abstract

**Background:**

Regarding the importance of children’s developmental status and various factors that delay their development, this study was conducted to examine the correlation between mothers’ depression levels and the developmental delay in infants.

**Methods:**

This descriptive study was performed on 1053 mothers and their infants’ age 6 to18 month-old in medical centers affiliated with Shahid Beheshti University of Medical Sciences, Iran, in 2014-2015. The participants were selected through multi-stage random sampling. The following instruments were used in this study: A demographic and obstetric specification questionnaire, infant specification questionnaire, the Beck Depression Inventory, and the Ages and Stages Questionnaire to determine the status of the children’s development. The data were analyzed using SPSS19 software, Mann-Whitney; independent T-test and logistic-Regression tests were used.

**Results:**

The results showed that 491 mothers (46.7%) suffered mild to extremely severe depression. The delay in infant development was 11.8%. The Mann–Whitney test showed a correlation between mothers’ depression levels and developmental delay in infants (P=0.001). Moreover, there was a significant correlation between mothers’ depression and developmental delays in gross-motor and problem-solving skills (P<0/05). In logistic model age of infants showed significant correlation with developmental delay (P=0.004 OR=1.07), but unwanted pregnancy, gender of infants, type of delivery and socioeconomic status had no correlation with developmental delay.

**Conclusion:**

Given the correlation between mothers’ depression and infant development, it is recommended to screen mothers for depression in order to perform early interventions in developmental delay.

## 1. Introduction

Despite advances in medical sciences in diagnosis and treatment of diseases, the developmental delay of infants is still considered one of the global health problems in developed and developing countries (de Moura et al., 2010). Developmental delay usually refers to children that do not display prominent developmental features that they are expected to for their age (Baker, 2001). After infections and trauma, developmental and behavioral problems are the most common in pediatric medicine (Behrman et al., 2004; Torabi et al., 2012).

It is estimated that approximately 200 million children worldwide do not enjoy a favorable development or cannot achieve it (de Moura et al., 2010). This problem is not equally prevalent worldwide and even developed countries account for high numbers. In at-risk populations, the rate for children with developmental disabilities has been reported up to the 30% (Soleimani, Vameghi, & Dadkhah, 2009; Amir Ali Akbari et al., 2012). Approximately 15%–20% of children in the United States suffer developmental or behavioral disabilities (Soleimani et al., 2009). This rate has been reported at 15% in Jamaica, 8% in Bangladesh, 15% in Pakistan, in India in different areas up to 1.5% to 2.5% in children under 2 years, 3.3% in Brazil, and 12.5% in Holland (de Moura et al., 2010; Poon et al., 2010; Potijk et al., 2013). In Iran, 18.7% in Isfahan, 22.5% in Qazvin, and 19.8% in Karaj have been identified (Torabi et al., 2012; Soleimani et al., 2013; Soleimani, Khoshbin, & Shams, 2001). Children’s delayed development is responsible for huge costs of diagnosis, treatment, and care. It is often a huge challenge in terms of time and energy, and economy and equipment for families and staff. It imposes costs of specialized schooling for these children, which is one of the problems for the education and health systems (Poon et al., 2010; First et al., 1994; Andersen et al., 2008).

The main cause of developmental disabilities remains unknown, and in some cases, a single factor cannot be blamed (Naddawi et al., 2013; Persha et al., 2007). It involves a wide range of causes and demographic factors. Children’s development is affected by prenatal, psycho-social, biological, hereditary, and environmental factors. In other words, human development is a continual and dynamic interaction of biological and acquired factors (de Moura et al., 2010). The first year in life is vitally important in the child’s development. Life outside the uterine and fetal existence together identifies a path on which growth and development of a person is formed, influenced by genetic, environmental, and social factors (Soleimani et al., 2001; Soleimani, 2007). Human development is a complex and massive issue. If growth is considered the enlargement of body size or different parts, development should be considered as functional changes that can be affected by the child’s environment. Because of the rapid changes in the first two years of life, studying dimensions of development in infancy is highly important (Soleimani & Karimi, 2005). Unfavorable maternal circumstances such as malnutrition, smoking, drug abuse, insufficient activity, and inadequate prenatal care can lead to unfavorable fetal growth, which is considered a health risk for a child’s future. Also, early experiences during childhood, because of the persistent flexibility of biological systems, are important in a child’s future health (Soleimani et al., 2009; NTRD, 1982). Studies indicate that unsuitable and abnormal conditions for the infant such as, mother’s marital status, tobacco or alcohol use during and after pregnancy, complications of pregnancy, labor, birth, and postpartum are the risk factors in developmental delay. Preterm delivery and low birth weight (especially less than 1500 g) are recognized as extremely important risk factors (Oelgado et al., 2007). Also, the mother’s psycho-social harms during and after pregnancy, low maternal socio-economic status, cultural issues, child abuse, parental psychological stress (especially the mother’s), environmental factors, and parents’ bad behaviors adversely affect fetal and neonatal development (de Moura et al., 2010; Soleimani & Karimi, 2005; Oelgado et al., 2007; Huggenberger et al., 2013; Conners-Burrow et al., 2009). Given that the occurrence of developmental delay usually involves more than one factor, identifying risk factors is highly important (Persha et al., 2007).

Children that live with depressed mothers have difficulties in social interaction and adaptations. Cortical levels and heart rates are higher in children of depressed mothers than in children of non-depressed mothers. Therefore, mothers’ mental health conditions not only affect their own health, it affects their infants’ health and development as well (Petterson et al., 2001). According to recent evidence, children’s delayed development is often associated with mothers’ stressful life events, anxiety, depression, stressful jobs, physical abuse, and low social support (Ceballo & McLoyd., 2002). Mothers’ psychological problems affect quality and quantity of childcare; a lack of a child’s attention to learning stimulants leads to his learning difficulties, it also leads to severe excitability of the child, which leads to behavioral problems (Propper & Rigg., 2007; Berg et al., 2003). Depressed mothers often cannot meet their child’s social and emotional demands, which lead to a series of restrictions and non-performance of maternal duties, causing cognitive problems, negative recognition and performance toward the child and other family members, leading to behavioral problems in the child (Ordway, 2011). Studies reveal that early and timely interventions to identify neonates and children at-risk prevent many complications following delayed development and disabilities in these children. Thus, identifying high-risk women and children, and beginning interventions before the problem occurs appears to be the best and most rational solution (Poon et al., 2010; Oelgado et al., 2007). We investigated correlation between mothers’ depression and developmental delay in Infants aged 6-18 months.

## 2. Material & Methods

This cross-sectional study was performed on 1053 mothers and their infants ranging from 6 to18 months-old in medical centers affiliated with Shahid Beheshti University of Medical Sciences from June 2014 to January 2015 in Tehran, Iran.

First, a list of clinics in the areas covered by that university was prepared. Then, some medical centers were randomly selected from each area, and a quota of participants from each center was determined considering the population covered by that center. In total, the studied participants were selected from 11 medical centers. Considering the 20% frequency of developmental disorders, the minimum sample size was calculated as 700 people, but was raised to 1000 people for the possible sample loss, and eventually, the information of 1053 people was collected.

The inclusion criteria were as follows: Iranian mothers of 18–35 years old, with a history of pregnancy fewer than four times, no medical conditions during pregnancy and the study, no history of problems such as preeclampsia, placental abruption, polyhydroamniuos, stillbirth, and newborns with congenital malformations, no history of any developmental disorders in their other children and in their relatives, no use of forceps or vacuum, and no complications such as bleeding and dystocia in their recent childbirth. The infants who were 6, 8, 12, and 18 months old, were born of a singleton pregnancy, had an Apgar score of greater than 7, were not premature (under 37 weeks) or post-term (more than 42 weeks), did not have fetal growth restriction, did not have a history of hospitalization, were over 2500 g at birth, were approved to have physical health by the center’s physician, received iron supplement, and did not have a growth delay.

Once mothers were briefed on the objectives of the study, and gave their consent to participate in the study, they were provided with the questionnaires. The instruments used for data collection included questionnaires of demographic, socioeconomic status, obstetric information, recent childbirth specification, infant specification, the Beck Depression Inventory for measuring the mothers’ depression levels, and the ages and stages questionnaire (ASQ) to determine the status of infant development. The validity and reliability of the demographic, socioeconomic status, obstetric questionnaire and infant specification questionnaire were determined using content validity and test-retest. (The correlation coefficient was 94%-97% from 10 checklists).

The Beck Depression Inventory-II (BDI-II) was used for screening of depression; it is the most commonly used measure of depression with 21 items scoring from 0 to 63. The scores 0–9, 10–18, 19–30, 31–40, and 41–63 respectively indicated normal, mild, moderate, severe, and extremely severe depression. Reliability of the test has been proved in different studies (Razavi et al., 2012; Jakšić et al., 2013; Hall et al., 2013). The internal consistency was confirmed with Cronbach’s alpha of 0.87 and reliability coefficient was found at 0.74 for the Iranian population (Ghassemzadeh et al., 2005).

ASQ is a reliable tool with Cronbach’s alpha of 0.86 and reliability of 0.93 for Iranian infants (Vameghi et al., 2013). It was used in several research (Torabi et al., 2012; Amir Ali Akbari et al., 2012; Soleimani et al., 2013). ASQ is a screening system with 19 questionnaires for the ages of 4, 6, 8, 10, 12, 14, 16, 18, 20, 22, 24, 30, 33, 36, 42, 78, 54, and 60 months. ASQ aims to distinguish the children in need of further development examinations from normal children. Each age group has 30 items, six for each of the five domains (namely communication, gross-motor, fine-motor, problem-solving, and personal-social skills). The items were designed in a way that parents with minimum literacy could understand and answer them by themselves. Each item has three options: “Yes” when the child can do the activity in the question completely, “Not yet” when the child has not done the activity in the question at that time, and “Sometimes” when the child can sometimes do the activity in the question. The answer “Yes,” “Sometimes,” and “Not yet” are respectively assigned 10, 5, and 0 points. The total score of the five domains is compared with the standard scores (cut-off points). If the infant’s score is lower than the defined limit, the infant should be examined more thoroughly and carefully. The infants who cannot gain the cut-off point score relevant to each domain has problems in that domain. The cut-off points are determined according to the guidelines of the Ministry of Health and Medical Education for Iranian children (Ministry of Health and Medical Education., 2005). All mothers and infants have problem were followed by specialist.

The data were analyzed with SPSS version 19 (Chicago, IL, USA). The data were analyzed using SPSS19 software, Mann-Whitney; independent T-test and logistic-Regression tests were used. The significance level was set as 0.05.

## 3. Results

The results showed that the mean age of mothers in the group with delayed development and mothers in the group with normal development was 29.32 ± 5.12 years and 29.95 ± 5.18 years, respectively. The mean age of infants was 10.58±4.48 months. The majority of mother in two groups were housewives (93.6%) with high school education (85.6%), the majority of infants were males (52.2%) and majority type of delivery was cesarean section (51.3%).

Furthermore, the results showed that 46.7% of the mothers suffered mild to extremely severe depression, and delayed infant development was 11.8%. The prevalence of developmental delay in 5 domains was showed in Table 1.

**Table 1:** Frequency of developmental delay in infant aged 6-18 mounths

Groups		

Domains of Development	Normal Development	Deley Development
	Frequency(percent)	Frequency(percent)
Communication	1033(1.98)	20(9.1)
Gross Motor	1030(8.97)	23(2.2)
Fine Motor	1014(3.96)	39(7.3)
Problem-Solving	1011(96)	42(4)
Personal-Social	992(2.94)	61(8.5)
Total	929(2.88)	124(8.11)

The Mann–Whitney test showed a significant correlation between mothers’ depression levels and developmental delay (P=0.001) (Table 2). Moreover, there was a significant correlation between mothers’ depression levels and developmental delays in gross-motor (P=0.001) and problem-solving skills (P=0.015) (Table 3).

**Table 2:** Correlation of mothers’ depression level and developmental delay

Level of Depression (Score)	Groups		

	Normal Development	Deley Development	Total
Frequency(percent)	Frequency(percent)	Frequency(percent)
**(9-0)** None	511(91.1)	50(8.9)	561(100)
**(18-10)** Mild	248(89.2)	30(10.8)	278(100)
**(30-19)** Moderate	125(80.1)	31(19.9)	156(100)
Sever(31-40) & Extremely Sever(41-36)	45(77.6)	13(22.4)	58(100)
Total	929(88/2)	(11/8) 124	1053(100)

Result of Mann-Withney test		P=0.001	

**Table 3:** Correlation of mothers’ depression and developmental delay (and 5 domains of development)

Domains Depression	Problem-Solving F(%)*	Delay	Fine F(%) Motor	Delay	Gross Motor F(%)	Delay	Communication F(%)	Delay	Personalsocial F(%)	Delay
	_	+	_	+	_	+	_	+	_	+
None	536(95.5)	25(4.5)	543(96.8)	18(3.2)	555(98.9)	6(1.1)	552(98.4)	9(1.6)	539(96.1)	22(3.9)
Mild	262(94.2)	16(5.8)	266(95.7)	12(4.3)	273(25.9)	5(1.8)	273(98.2)	5(1.8)	267(96.0)	11(4.0)
Moderate	142(91.0)	14(9.0)	150(96.2)	6(3.8)	148(94.9)	8(5.1)	151(96.8)	5(3.2)	149(95.5)	7(4.5)
Sever& Extremely Sever	52(89.7)	6(10.3)	55(94.8)	3(5.2)	54(93.1)	4(6.9)	57(98.3)	(1.7)1	56(96.6)	2(3.4)
Total	992(94.2)	61(5.8)	1014(96.3)	39(3.7)	1030(97.8)	23(2.2)	1033(98.1)	20(1.9)	1011(96.0)	42(4.0)
Result of Man-Witney test	P=0.015		P=0.379		P=0.001		P=0.368		P=0.898	

* F (%) =Frequency (percent).

In logistic model age of infants showed significant correlation with developmental delay (P=0.004 OR=1.07), but unwanted pregnancy, gender of infants, type of delivery and socioeconomic status had no correlation with developmental delay.

## 4. Discussion

This study revealed a correlation between mothers’ depression levels and infant development. Mothers’ depression status is a risk factor for social, psychological, and cognitive development (Darcy et al., 2013). Women are at risk of postpartum depression, a state experienced by about 15% to 20% of women. The mild to severe depression in the first six months after the childbirth is observed in 10% to15% of women (Gaynes et al., 2005; O’Hara, 2009; Vesga-Lopez et al., 2008). Sometimes, the symptoms of depression appear, but are not diagnosed. Women experiencing postpartum depression may suffer depression in the future by 50% to 62%. Given that children regularly communicate with their mother, a mother’s state of depression affects the mother-child relationship. These children experience their mother’s anxiety, are not supported by their mothers in the effort to do activities, and cannot adapt to different situations. These children have trouble communicating with their friends and playing with them. Children of depressed mothers are at higher risk of depression and anxiety than other children (Petrozzi et al., 2013). Mothers’ depression levels may affect their own lives, people living with them, and those who are in contact with them. In the first year of life, children are influenced by their parents’ actions, especially their mother’s behaviors, and their cognitive emotional development depends on it. Mothers’ depression levels can affect the normal development of a child because of the impact of depression on a mother’s abilities within the maternal role and the accompanied higher risk of mental, cognitive, and attention disorders in children (Edward et al., 2012). Studies show the effect of depression on parent–child relationship. When parents have relationship problems, their children have the same problems too (van der Toorn et al., 2010). Servili et al studied the development of 1-year-old children with depressed mothers the children had problems with cognitive and social domains of development. Although there was no significant relationship between mothers’ depression levels and child development, children’s scores concerning gross motor skills were lower than that of children with non-depressed mothers (2010). Black et al. showed mood problems in children living with depressed mothers (2007). Santos et al. found a correlation between mothers’ mental disorders and their children’s mental disorders. The parents’ mental health and parent–child relationship are important determinants of children’s mental health (2014). Toth et al. conducted a study on the effect mothers with severe depression have on their children and compared 63 children of depressed mothers with 68 children of non-depressed mothers. They showed that mothers’ depression levels influenced children’s accountability and relationships (2009).

Darcy et al studied the effect of mothers’ depression levels on the quality of life and health of children aged 4–6 months and showed that these children had a lower quality of life and state of health than other children; they showed a significant relationship between the mothers’ symptoms of depression and the children’s physical health. Moreover, there was a significant connection between mothers’ depression and children’s quality of life and mental health. However, Darcy did not find a relationship between mothers’ depression levels and child development (2011). Shonkoff et al explained that mothers’ depression levels and personality disorders increased the stress responses in children, and this resulted in developmental disorders in children. Moreover, the risk of behavioral disorders, such as drug abuse, heart diseases, and diabetes increased in such children (2012). Depressed mothers show negative parental behaviors, such as neglecting a child, or performing unpredictable behaviors with the child. These mothers also show fewer reactions to their child, which is a risk factor within the development of the mother–child relationship; there was significant relationship between mothers’ depression levels and child development at the age of 15 months (Goodman et al., 2009). Children of depressed mothers have problems with the management of uncontrollable events (Crockenberg et al., 2008). Petterson et al showed the effects of mothers with depression on their male and female children’s development (2001). Dawson et al reported that the electroencephalography revealed abnormalities in the frontal lobe of the brain in children of depressed mothers, as these abnormalities were associated with behavioral disorders at the age of 3 years (2003). These children showed lower activity in the frontal and parietal lobes of the brain. Electroencephalography in children of depressed mothers showed less activity in the left frontal lobe of the brain while they were playing and less activity in the right frontal lobe of the brain while they were in special conditions, such as separation from the mother (Dawson et al., 1992). Koutra et al., showed the relationship of mothers’ depression with delayed development of children aged 18 months in the gross motor and cognitive domains of development (2013). Moses et al also revealed a relationship between mothers with depression and the development of children, especially in the cognitive domain (2004).

Therefore identifying women at risk of depression and beginning medical interventions before the event seems the best solution, early and timely interventions to identify at-risk infants and children are much more effective than many outcomes that follow delayed development and children’s disabilities.

## 5. Conclusion

Currently, there is no medical treatment to completely stop the progress of delayed development. Thus, Because of the huge number of problems caused by having a child with developmental delay, early diagnosis and timely referral is highly important and can have the most benefit for children with developmental disabilities and their families. Thus, monitoring a child’s development and screening for such problems in every visit, especially the child’s first visit, is necessary for health examinations.
